# A Preliminary Report on the Largest Ongoing Outbreak of Lead Toxicity in Iran

**DOI:** 10.1038/s41598-020-64859-8

**Published:** 2020-07-16

**Authors:** Nasim Zamani, Omid Mehrpour, Hossein Hassanian-Moghaddam, Maryam Jalali, Alireza Amirabadizadeh, Saeed Samie, Shahram Sabeti, Ali-Asghar Kolahi

**Affiliations:** 1 Social Determinants of Health Research Center, Shahid Beheshti University of Medical Sciences, Tehran, Iran; 2 Department of Clinical Toxicology, Loghman Hakim Hospital, School of Medicine, Shahid Beheshti University of Medical Sciences, Tehran, Iran; 3 Medical Toxicology and Drug Abuse Research Center (MTDRC), Birjand University of Medical Sciences, Birjand, Iran; 4 Rocky Mountain Poison and Drug Center, Denver, Colorado USA; 5 Clinical Biochemistry Founder and Director of Noor Pathobiology Lab, Tehran, Iran; 6 Department of Biostatistics, Birjand University of Medical Sciences, Birjand, Iran; 7 Pars Advanced and Minimally Invasive Medical Manners Research Center, Pars Hospital, Iran University of Medical Sciences, Tehran, Iran; 8 Department of Pathology, Loghman Hakim Hospital, Shahid Beheshti University of Medical Sciences, Tehran, Iran

**Keywords:** Health policy, Diagnostic markers, Health policy, Diagnostic markers

## Abstract

No countrywide data exists on the patients’ characteristics of lead exposure in Iran. We aimed to evaluate the demographic characteristics and blood lead level (BLL) of these patients in the country scale during five consecutive years, including the epidemic outbreak year (2016). Between 2014 and 2018, records of all patients who had referred to two reference laboratories in Tehran, Iran, to check BLL were evaluated. Of 58,642 patients, 48,589 were male. Mean age was 44.9 ± 20.7 years. Males had higher BLLs and were significantly older. Median BLL was 16 µg/dL (0.3 to 263 µg/dL). Median BLL was significantly higher in 45- to 60-year-old patients. The highest median BLL was reported in May 2016 confirming our records about the peak of the epidemic. Although the frequency of high BLL declined after 2016, it never returned to the measures before that. Considering the ongoing high prevalence of increased BLLs after 2016 and similar environmental and occupational exposures as before, lead-contaminated opium still seems to persist in the Iranian opium black market. Substitution of this lead-contaminated opium by Opioid Maintenance Therapy (OMT)-prescribed opium tincture is recommended.

## Introduction

Heavy metal poisoning has been an issue in ancient countries, including Iran^1^. Lead can be easily absorbed inhalationally and/or through gastrointestinal route especially in children^2,3^. Most of lead is stored in cortical bones, teeth, brain, liver, and kidneys^4^. Signs and symptoms of lead exposure relative to the blood lead level (BLL) may widely vary based on the individual characteristics. In acute cases of toxicity, abdominal pain, anemia, constipation, muscular and skeletal pain, headache, anorexia, decreased libido, dizziness, short-term memory loss, nephropathy, and wrist/ankle drop may be present^3^. Lead encephalopathy is the most serious manifestation that may lead to permanent neurological sequelae and death^5^. In chronic cases, hypertension may develop^6,7^.

Environmental lead poisoning is an endemic problem in Iran^2^ due to occupational and environmental exposures. Substitution of lead with methyl tert-butyl ether (MTBE) in gasoline decreased the environmental contamination by gasoline in this country. On the other hand, it seems occupational lead toxicity has decreased in Iran over the recent years^8^. However, environmental contamination of the soil, water, vegetables and traditional herbal remedies by lead is still a concern^2,9,10^. It is supposed that increasing the number of lead processing industries affecting surface, drinking and ground water, rivers and seas (waste discharges) is a major source of environmental pollution^10^. Besides, illicit drugs contaminated with lead are a new health threat in recent decades^10^. Therefore, we expect persistent endemic lead exposure in Iran.

During the recent decade, opium impurities and lead-contaminated opium were recognized as a possible cause of lead poisoning in Iran^3,11^. After recognizing this health threat, some studies evaluated lead-contaminated illicit drugs in Iran. Adulterants were evaluated in samples of commonly abused psychoactive drugs including opium, Iranian crack, ecstasy tablets, and crystal methamphetamine^12^. It was shown that psychoactive drugs were adulterated with tramadol, ketamine, methadone, acetaminophen, and caffeine. Lead was detected in all analyzed samples, ranging from 9–90 ppm with the lowest amount detected in methamphetamine samples, while Iranian crack and opium samples had the highest contents of lead. In another study, heavy metals of illegal opioids samples (Iranian crack, heroin, and opium) were evaluated^13^. The authors concluded that heavy metals including lead existed in all samples. The highest lead (Pb; 138.1  ±  75.0 μg/g) and chromium (Cr; 447.4  ±  20.3 μg/g) contents were detected in opium samples. Heroin and Iranian crack samples had 48.8 μg/g and 30.8 μg/g lead, respectively.

This was again an endemic problem in Iran until 2016 when an epidemic of lead poisoning due to lead-contaminated opium peaked^14^. Studies during this period mentioned the possibility of existence of more than 42,000 lead-poisoned patients^15^. However, no official statistics were reported by Iranian Ministry of Health (MOH) regarding the number of the patients and their characteristics. This is while many of the patients were being checked for blood lead levels (BLLs) by limited reference laboratories equipped with the atomic absorption technique. Although blood was taken for BLL check in many cities, samples were transferred to two reference laboratories in the capital city. This study aimed to determine the basic characteristics and mean BLL of the patients during a five-year period of the study including the year of the epidemic outbreak to see any changes in the Iranian BLL trend in the country.

## Materials and Methods

There are two active reference laboratories in Tehran, Iran, that are equipped with atomic absorption technique to check for heavy metal poisonings. During the epidemic, patients with probable signs and symptoms of lead poisoning were referred, or their blood sample was taken and sent to these laboratories for BLL check.

Venous blood specimens obtained in EDTA tubes using lead-free equipment were used for analyzing blood lead. EDTA tubes containing less than half of the amount of blood required and/or with visible clots were discarded. All specimens were kept at room temperature and checked within 24 hours of specimen collection. All processes of sample preparation were performed in Class B2 laminar flow biological safety cabinets, to avoid contamination with environmental lead, if any.

The instrument calibrations were carried out according to the specifications of the manufacturer.

Two Quality control samples at clinically significant blood lead levels have been analyzed at each analytical run and when instrumental/operational problems have been detected.

The laboratories have participated regularly in external quality assessment programs provided by Pishgam Iranian QA system.

The available data of the patients admitted to these two laboratories (Nour and Pars reference laboratories) between 2014 and 2018 were retrieved and evaluated. Those for whom BLL had been checked were enrolled. Duplicate cases in each year were eliminated from analysis using ID and national code. These two laboratories were measuring almost all of blood lead samples in the country.

Our institutional review board (IRB) committee waived informed consent for retrospective studies while participants were anonymous. Data including patients’ demographic characteristics (age and gender), season of the year, and their BLL was recorded in a self-made sheet designed for the study. BLLs were categorized into three groups of BLLs < 10 (currently acceptable level), 10 < BLL < 50 (mildly elevated BLL), and >50 µg/dL (high BLL) and compared between different sex and age groups in five years. The data was then extracted into Statistical Package for Social Sciences (SPSS) software version 22 and analyzed using independent t-test, Mann Whitney U test, Kruskal-Wallis, and Duncan’s tests. Median (interquartile range; IQR) or mean ± standard deviation (SD) was used for description of quantitative variables with non-normal and normal distribution respectively. Frequency (%) was mentioned for qualitative variables. P values less than 0.05 were considered to be statistically significant.

### Ethics approval and consent to participate

This study was supported by Shahid Beheshti University of Medical sciences, Tehran, Iran, and approved by its ethics committee (IR.SBMU.RETECH.REC.1399.165). All methods were performed in accordance with the relevant guidelines and regulations.

## Results

**Figure 1:**
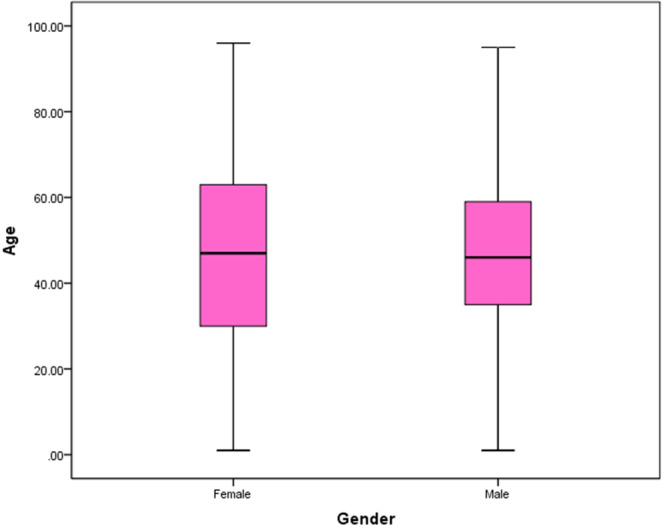
The median age of the subjects studied by gender.

Of the 58,642 patients whose BLL had been checked by these two reference laboratories, 48,589 (82.9%) and 10,053 (17.1%) were male and female, respectively. Mean age (all patients) was 44.9 ± 20.7 with median 46.0 years, males 45.7 ± 19.4 with median 46.0 years, and females 41.2 ± 25.9 with median 47.0 years (range; 1 to 96 years). The independent t -test showed that males were older than females (t = 19.5; P < 0.001) (Fig. 1).

**Figure 2:**
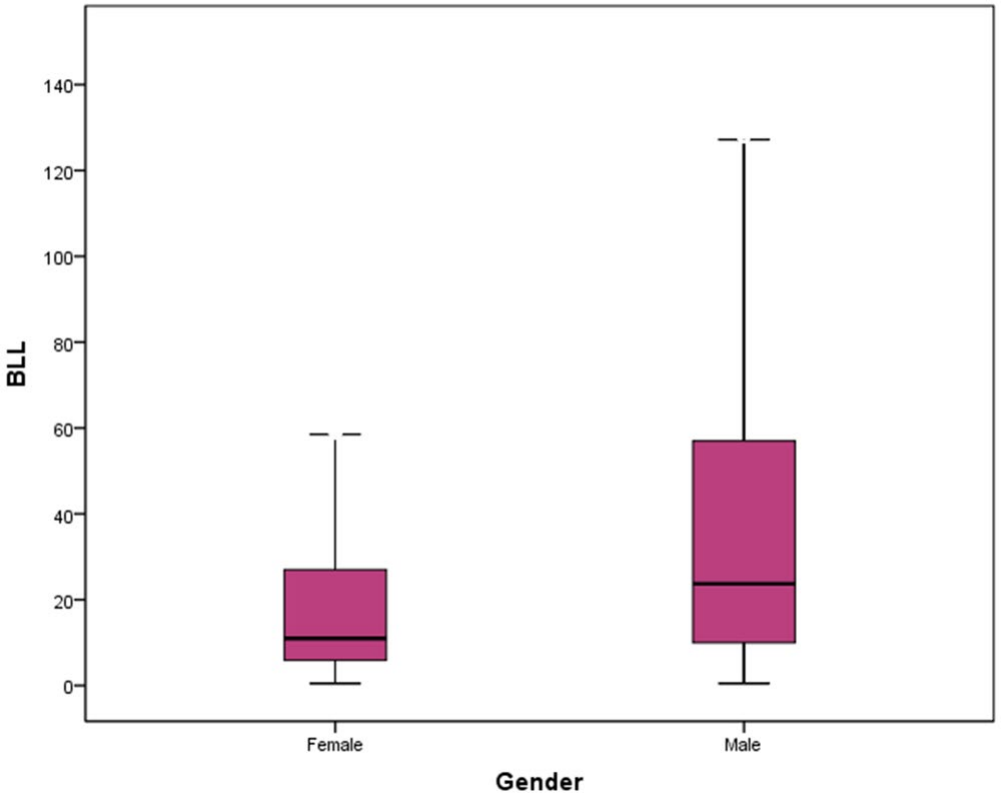
The median BLL of the subjects studied by gender.

Median BLL [Interquartile range; IQR] was 16 [8.4, 39] µg/dL with mean 35.85 ± 53.29 µg/dL in all patients (range; 0.3 to 263 µg/dL). Median BLL was 18 and 10 µg/dL in males and females (Fig. 2). Mann Whitney U test showed that median BLL was higher in males (z = 4834, P < 0.001). 18,369 (31.3%), 29,401 (50.1%), and 10,872 (18.5%) patients had BLLs less than 10, 10-50, and over 50 µg/dL, respectively. 27.6% of the males and 49.4% of the females had BLLs < 10 (currently acceptable range). Median BLLs of the patients in different age groups are shown in Table 1.

**Table 1:** Median BLL in different sex and age groups and different months of the year.

Variable	BLL (µg/dl) Median [IQR]	Mean ± SD	Test result
**Gender**			
Male	18.0 [9.0–42.2]	21.04 ± 45.95	z = 48.34 P < 0.001
Female	10.0 [6.1–20.0]	13.26 ± 25.87	
**Age group (years)**			
<15	8.7 [5.6–15.0]	12.41 ± 16.89	Χ2 = 346.65 P < 0.001
15–30	11.0 [7.0–25.7]	17.55 ± 29.81	
30–45	16.0 [8.2–45.0]	24.84 ± 46.51	
45–60	21.8 [10.0–48.0]	30.06 ± 49.98	
60–75	20.0 [10.3–38.0]	29.91 ± 40.83	
>=75	15.0 [9.0–29.0]	21.44 ± 33.14	
**Years**			
2014	7.0 [2.4–21.0]	8.01 ± 28.34	Χ2 = 537.38 P < 0.001
2015	6.5 [2.5–20.0]	7.21 ± 24.86	
2016	25.2 [10.0–59.0]	43.51 ± 42.33	
2017	15.0 [9.0–31.9]	19.81 ± 33.62	
2018	11.8 [7.8–24.0]	14.67 ± 30.63	
**Month**			
January	13.3 [7.6–32.0]	19.56 ± 18.13	4
February	15.0 [8.0–36.7]	22.48 ± 43.36	
March	16.0 [8.5–39.0]	24.37 ± 42.37	
April	17.5 [8.8–43.1]	16.07 ± 46.51	
May	22.2 [9.5–59.0]	30.68 ± 63.52	
June	19.0 [9.6–46.0]	26.72 ± 31.73	
July	15.9 [9.0–38.0]	22.79 ± 43.30	
August	16.0 [8.7–36.0]	24.57 ± 39.03	
September	16.0 [9.0–36.3]	24.35 ± 40.08	
October	14.5 [8.5–35.2]	20.40 ± 36.50	
November	13.7 [7.0–33.0]	19.40 ± 35.20	
December	13.0 [7.0–30.0]	19.04 ± 31.68	

**Figure 3:**
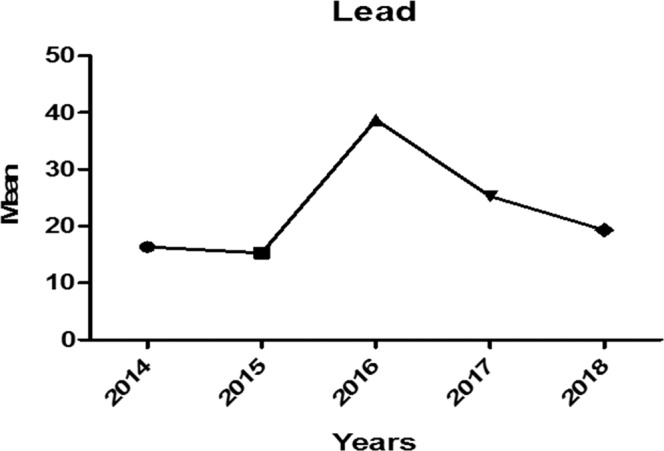
Trend of Lead poisoning in Five Consecutive Years Including the Epidemic Outbreak Year.

**Figure 4:**
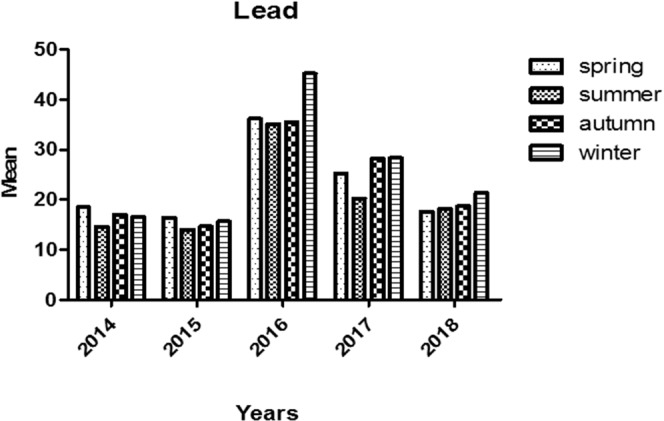
Trend of Lead Poisoning in Different Seasons during the Study Period.

Duncan’s test showed that median BLL was significantly higher in 45- to 60-year-old patients compared to all other age groups (P < 0.001). Table 2 shows the BLLs in different age groups. Figures 3 and 4 show the trend of high BLLs in five consecutive years of the study and its distribution in different seasons and years.

**Table 2:** Number of the Patients in Each BLL Group Based on their Age, Gender, and Year of the Study.

	Blood lead level (µg/dl)		
	<10	10–50	>=50
**Gender**			
Male	13,399 (27.6%)	25,247 (52.0%)	9,943 (20.5%)
Female	4,970 (49.4%)	4,154 (41.3%)	929 (9.2%)
**Age group(years)**			
<15	3,427 (57.3%)	2,056 (34.4%)	495 (8.3%)
15-30	2,286 (42.7%)	2,354 (44.0%)	711 (13.3%)
30-45	5,176 (32.0%)	7,440 (46.0%)	3,571 (22.1%)
45-60	3,856 (24.1%)	8,300 (51.9%)	3,849 (24.0%)
60-75	2,516 (22.3%)	6,900 (61.2%)	1,865 (16.5%)
>=75	1,108 (28.9%)	2,351 (61.2%)	381 (9.9%)
**Years**			
2014	912(57.1%)	544 (34.1%)	141 (8.8%)
2015	2,295 (60.3%)	1,233 (32.4%)	275 (7.2%)
2016	5,469 (22.8%)	11,138 (46.4%)	7,404 (30.8%)
2017	5,331 (28.6%)	10,900 (58.4%)	2,418 (13.0%)
2018	4,362 (41.2%)	5,586 (52.8%)	634 (6.0%)

The highest median BLL was reported to be 25.2 µg/dL in 2016 (epidemic year), and the least BLL was reported in 2015 (median of 6.5 µg/dL; Fig. 3). Also, the highest BLL has been reported in May, which confirms our records of the peak of the epidemic outbreak in May 2016. In general, mean BLL was highest in winter months 43.3 ± 85.9 (median [IQ range]: 19.7 [1.0–66.0] µg/dL) and lowest in summer months 31.2 ± 35.5 (median [IQ range]: 13.9 [7.8–45.0] µg/dL; P < 0.001).

## Discussion

Because opium is often sold based on weight, lead may be added to it during manufacturing or at the time of packing in body packers to boost its weight. This may result in an intoxicating level of lead in opium samples. Lead can also be simply a non-purposeful impurity induced during refining process of production^16,17^. Inorganic lead poisoning due to lead-contaminated heroin abuse was reported in 1989^18^. Lead-adulterated marijuana and methamphetamine are also reported^19–21^. In some areas of the US, illegally distilled alcohol (moonshine) is a significant source of lead exposure^22^. Chia *et al*.^23^ published the first report of lead poisoning due to opium in a 40-year-old Chinese woman. After that, several reports were published from other parts of the world including Australia^24^, England^25^, Netherlands^26^, and Scotland^27,28^.

The higher number of male patients compared to the females seems a predictable finding as males are more engaged in high-risk occupations, use opium more commonly, and are more prone to be environmentally exposed to lead^14^. However, almost one-fifth of our patients were females. Considering the fact that the patients have been referred with the possible lead poisoning, this may show a rise in the number of female opium users or simply due to an increase in the number of female opium users coming forward for testing.

Higher BLL in older patients also emphasizes on occupational/environmental cumulative effect of lead and the fact that patients with a history of longer opium use/abuse are more prone to lead poisoning. Although the history of amount and period of dependency is not clear, older patients have presumably been exposed to contaminated opium for more years, making their dependency heavier due to the possible increasing tolerance to the opium during years of abuse, and have a higher BLL.

The currently acceptable value of lead has recently been reduced from 25 to 10 µg/dL by Centers for Disease Control and Prevention (CDC). However, it has been recommended to be even less in pregnant women and children^29,30^. However, as revealed by our evaluations, mean BLL is further higher in our sample. Although this sample does not represent the total population in our country, it well shows the higher possibility of lead contamination in our people.

Higher BLLs in 2016, in the context of more references to the emergency departments throughout the country which confirmed lead-contaminated opium as the source of lead^13,31^, confirms the epidemic of lead-contaminated opium use in our society as the environmental and occupational factors have not changed during the whole period of the study^30^. Although the frequency of high BLL has decreased after 2016 (epidemic year), this has never returned to its baseline measures. This may show the possibility of an ongoing epidemic of lead-contaminated opium, which may be managed by substitution of clean opium tincture provided by opioid maintenance therapy (OMT) clinics instead of the lead-contaminated opium in the black market.

As mentioned, our sample is not representative of our whole society. However, the two reference laboratories with atomic absorption technique have undoubtedly covered most of the patients suspected to be lead-poisoned as all centers visiting patients with possible lead poisoning referred their patients to one of these two referral laboratories for BLL check. Thus, the current data can, more or less, represent the features of lead poisoning in Iran right now. Although the precise number of patients and treatments given to them were not recorded and published by Iranian Ministry of Health, the number of antidotes used throughout the country showed an estimated number of more than 16,000 treated and more than 40,000 untreated patients who were managed by discontinuation of oral opium use due to limitations in antidote availability. The authors suspect that this increase in numbers of people recognized as suffering from lead poisoning will put an increased burden on the Iranian health services. The authors would support further review of the situation by health authorities. This made the authorities think about antidote stock piling strategies. Our available data is limited which is mainly due to the retrospective nature of the study. On the other hand, acceptable BLLs have recently been reduced to less than 5 µg/dL in children while we could determine that the median BLL of children less than 15 years old was more than this limit (8.7 µg/dL) and almost 42% of children had a blood lead level more than 10 µg/dL that could affect their growth and might cause neurodevelopmental disabilities. More prospective studies on characteristics of endemic lead poisoning due to lead-contaminated opium and examination of the wide strategies applied for epidemic and antidote management in Iran are warranted.

We believe that not all potential patients (those who had been asked to check their BLLs) referred to check for their BLLs, and this is probably the most important limitation of the current study. On the other hand, only patients with more severe signs and symptoms may have referred to check their BLLs which is another possible limitation and bias source. Moreover, we did not have access to other laboratory parameters associated with lead toxicity like hemoglobin (normochromic and normocytic or hypochromic and microcytic anemia), and basophilic stippling cells^32,33^.

## Conclusion

Lead-contaminated opium might be a significant cause of high BLLs and possible lead toxicity in Iran. Although the frequency of high BLLs has declined after 2016 (epidemic year), it has not been returned to its baseline measures in 2015 which may be due to contamination of lead in the opium black market or other possible resources of lead contamination. Appropriate measures should be carried out to encourage opium addicts to quit opium use or replace it with opium tincture provided by OMT clinics.
